# Morphophysiological mechanism of rice yield increase in response to optimized nitrogen management

**DOI:** 10.1038/s41598-017-17491-y

**Published:** 2017-12-08

**Authors:** Wei Zhou, Tengfei Lv, Zhiping Yang, Tao Wang, Yong Fu, Yong Chen, Binhua Hu, Wanjun Ren

**Affiliations:** 1 Key Laboratory of Crop Eco-physiology and Farming System in Southwest China, Ministry of Agriculture, P. R. China, Sichuan Agricultural University, Chengdu, Sichuan 611130 China; 2 Institute of Eco-agriculture, College of Agronomy, Sichuan Agricultural University, Chengdu, Sichuan 611130 China; 3 Rice Research Institute, Sichuan Agricultural University, Chengdu, Sichuan 611130 China

## Abstract

The yield-increasing mechanism of an optimized nitrogen fertilizer application (OFA) in rice was reported in this work through a three-year test. Results showed that the number of branches and spikelets increased, panicle length, the diameter and vascular bundle number of panicle-neck internode improved with OFA. Under the condition of OFA, high effective leaf areas, especially for the flag and the second upper leaf areas, increased, the net photosynthetic rate of the upper three leaves promoted, so the photosynthetic productivity went up by a large margin; moreover, the content of soluble protein and chlorophyll of leaf also increased, and the content of soluble sugar and malondialdehyde (MDA) decreased, as a result in slowing down the senescence speed in leaves, and increasing the photosynthetic time. Gene expression level, including *MOC1, LAX1, SP1, GS1;1*, were up-regulated obviously in different panicle initiation stage under OFA condition, which conduced to the increase in the secondary branches and spikelets. So we concluded that the changes in organ formation and panicle structure, together with the responses in physiological and molecular made the photosynthetic area, rate and time all increased with OFA, which provided the matter basis for the big panicle development, consequently, got a higher yield.

## Introduction

Over the centuries, rice (*Oryza sativa* L.) has been the major source of calories for a large proportion of the world’s population. Since the first green revolution in the 1960s, the preference was given to high-yield semi-dwarf modern rice varieties^1^ to satisfy the demands of the rapidly increasing population. Meanwhile, because of the improved lodging resistance of semi-dwarf and dwarf rice varieties, the fertilizer application rate, especially that of nitrogen fertilizer, have been increasing rapidly. Heffer^2^ reported that about 15% of total nitrogen fertilizers used in agriculture is applied to rice alone. However, the excessive fertilization and unique conditions of paddy fields promote nitrogen losses to the environment, which results in low utilization rate and unstable grain production. In the past few years, many optimized nitrogen management strategies have been put forward and tested for field production, such as site-specific nitrogen management, integrated soil-crop system management, topdressing, and others^3–7^. A core technology implemented in those strategies to increase rice yield and nitrogen use efficiency is split fertilization, especially topdressing, applied during the panicle initiation stage.

Nitrogen is one of the key nutrients that limit crop growth and yield potential of cereals in many production systems. Studies have shown that application of nitrogen fertilizer at the young panicle differentiation stage could increase the number of spikelets significantly^5,8^. There is a close relationship between the spikelet number and the amount of nitrogen accumulated by the late spikelet differentiation stage^9,10^. However, topdressing time and frequency of fertilization varies widely among the nitrogen management strategies, and farmers cannot easily or conveniently estimate topdressing time. On the basis of previous research and the experience gained during the many years of practical production, we put forward an optimized nitrogen fertilizer application (OFA)^11^ appropriate for the *indica* hybrid rice, which is cultivated over a wide area and plays an important and irreplaceable role in rice production system in China. The OFA is a four-step fertilization method: nitrogen fertilizer is applied at one day before transplantation, seven days after transplantation, jointing stage, and 15 to 20 days after the jointing stage. Farmers can easily identify the jointing stage, and therefore, the topdressing time of OFA can be easily selected without any instruments or assays. In addition, our previous studies have demonstrated that OFA increases nitrogen use efficiency and significantly improves rice yield by increasing the number of spikelets without increasing the nitrogen fertilizer application rate^11,12^.

The number of filled spikelets at the mature stage is a consequence of the differentiation, degeneration, and setting percentage of spikelets. Studies have shown that nitrogen fertilizer applied at the young panicle differentiation stage increases spikelet number^5,8^, but the mechanisms underlying this increase remain largely unknown. In addition, the number of spikelets is always negatively correlated with the setting percentage; hence, nutrient supply in the filling stage is very important for increasing the number of filled spikelets. Nutrient sources needed to fill spikelets are obtained mainly from two resources: one is dry-matter production before heading and the other is photosynthates in functional leaves after heading. Zhai *et al*.^13^ confirmed that dry-matter production after heading and its synchronization with grain filling are the key approaches to increase rice yield. Accordingly, we hypothesize that the OFA strategy can increase the number of spikelets remarkably, improve the production capacity of functional leaves after heading, and promote matter transport from source to sink, therefore increase rice production.

## Results

### Panicle phenotypic change and molecular regulation

**Figure 1:**
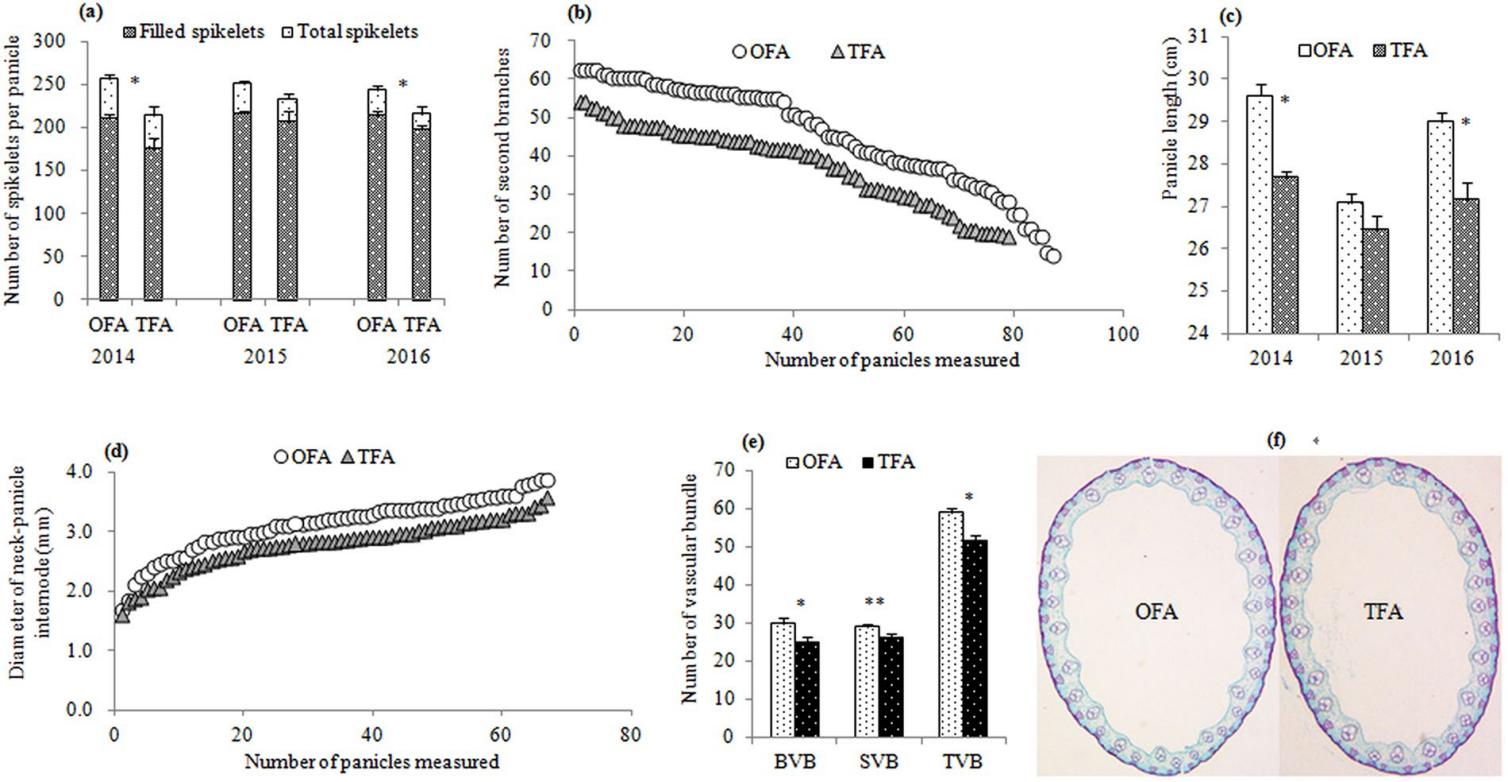
Phenotypic changes and morphological response to different nitrogen applications. (**a**) number of spikelets per panicle; (**b**) number of secondary branches; (**c**) panicle length; (**d**) diameter of the panicle-neck internode; (**e**) number of vascular bundles. BVB: large vascular bundle; SVB: small vascular bundle; TVB: total vascular bundle. (**f**) cross-section of vascular bundles. OFA: optimized nitrogen fertilizer application; TFA: traditional nitrogen fertilizer application; * and ** indicate significant difference of the two nitrogen treatments at 0.05 and 0.01 probability levels, respectively.

Morphological and structural features of panicles, including the number of filled and unfilled spikelets and secondary branches, panicle length, and diameter and number of vascular bundles of the neck-panicle internode were determined to identify phenotypic changes after the OFA. Nitrogen application had a significant effect on the number of branches and spikelets in rice panicle. The number of total spikelets in OFA treatments was higher by 6.8–20.2% compared to that in treatments with traditional nitrogen fertilizer application (TFA) (the difference was significant in 2014 and 2016) and contributed to the 3.7–19.7% increase in the number of filled spikelets at the mature stage (Fig. 1a). Figure 1b shows a significant increase in the number of secondary branches in plants after OFA, which was, on average, 20.5% higher than that in TFA treatments. The panicle after OFA treatment was longer than that after TFA treatment in the three years of experiment; the difference was significant in 2014 and 2016 (Fig. 1c). The dry matter produced before or after heading only go through the neck-panicle internode can be transported to panicle for grain filling. The diameter of the neck-panicle internode ranged from 1.60 to 3.57 mm in TFA and from 1.67 to 3.86 mm in OFA treatments, on average, the OFA increased the diameter of the neck-panicle internode by 12.23% compared to that reported for TFA treatments (Fig. 1d). Similarly, as reported in another study^14^, the number of spikelets per panicle was positively related to the neck internode diameter. Vascular bundle, as a part of the transport system, transports photoassimilates and moves water and nutrients, reflecting the capacity of rice ear for transporting nutrients during the grain-filling stage^15^. The results of the present study also revealed that the number of large and small vascular bundles, as well as the total number of vascular bundles, in the neck-panicle internode were significantly greater in plants after the OFA than after the TFA (Fig. 1e,f). The changes in phenotypic features demonstrated that the OFA promoted the formation of large panicles.

**Figure 2:**
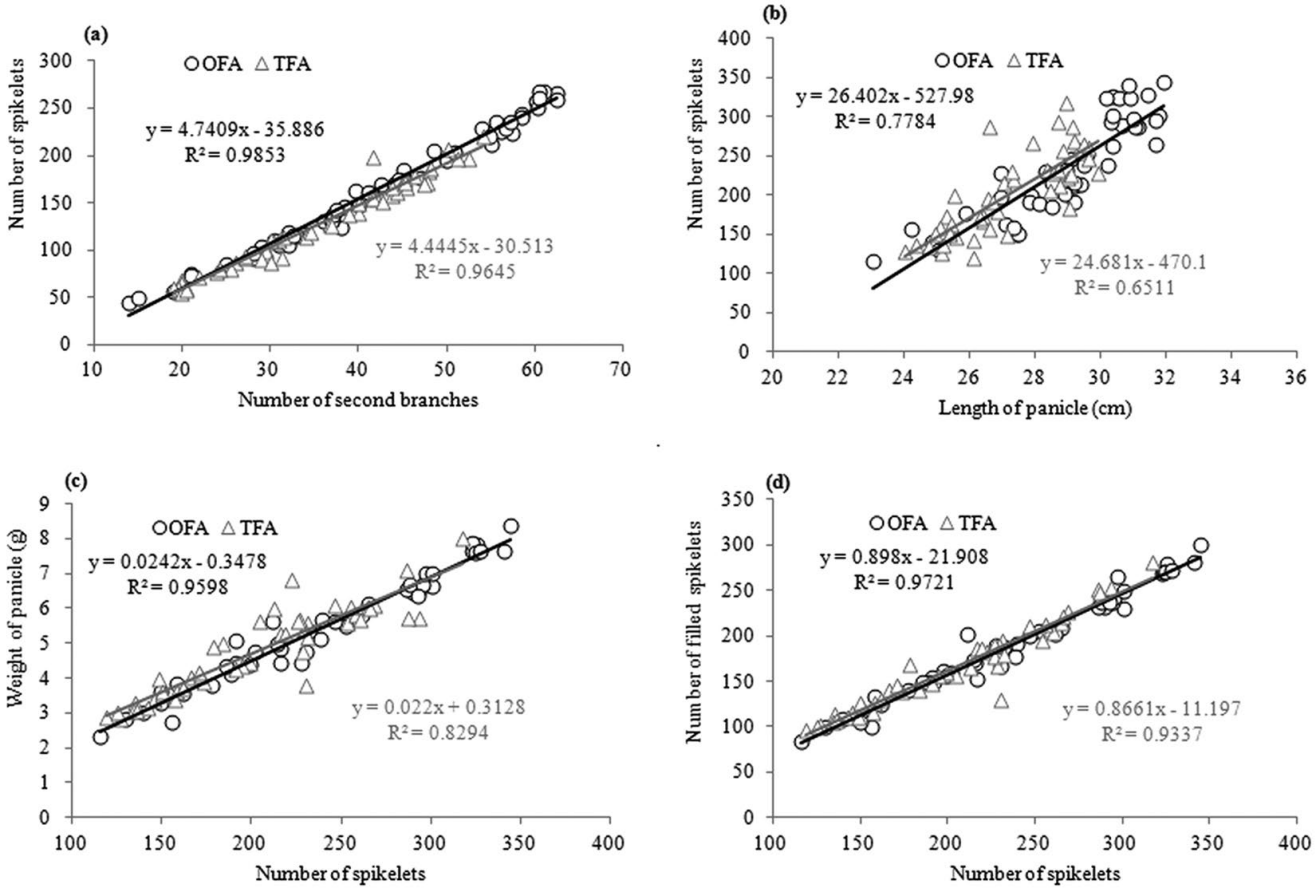
Correlation between the number of spikelets and panicle type. (**a**) correlation between the number of spikelets and the number of branches; (**b**) correlation between the number of spikelets and length of panicle; (**c**) correlation between the number of spikelets and the weight of panicle; (**d**) correlation between the number of spikelets and the number of filled spikelets. OFA: optimized nitrogen fertilizer application; TFA: traditional nitrogen fertilizer application.

Rice sink is determined by the number of spikelets and their weight. Since grain weight is genetically relatively stable, the number of spikelets is the primary determinant of the capacity of rice sink^16^. Many researches proved that nitrogen topdressing is highly effective in maximizing spikelet production^5,8^, and similar results were found in our study. The panicle length and the number of secondary branches are important predictors of spikelets. Thus, the number of spikelets was significantly and positively correlated with panicle length and the number of secondary branches. The linear equation slopes under OFA conditions were higher than that under TFA conditions (Fig. 2a,b), indicating that the increase in panicle length and number of branches provided more space for spikelets development and increased spikelet density per panicle. In addition, the number of filled spikelets and the weight per panicle were positively correlated with the number of spikelets (Fig. 2c,d); therefore the increase in the number of spikelets improved rice yield.

**Figure 3:**
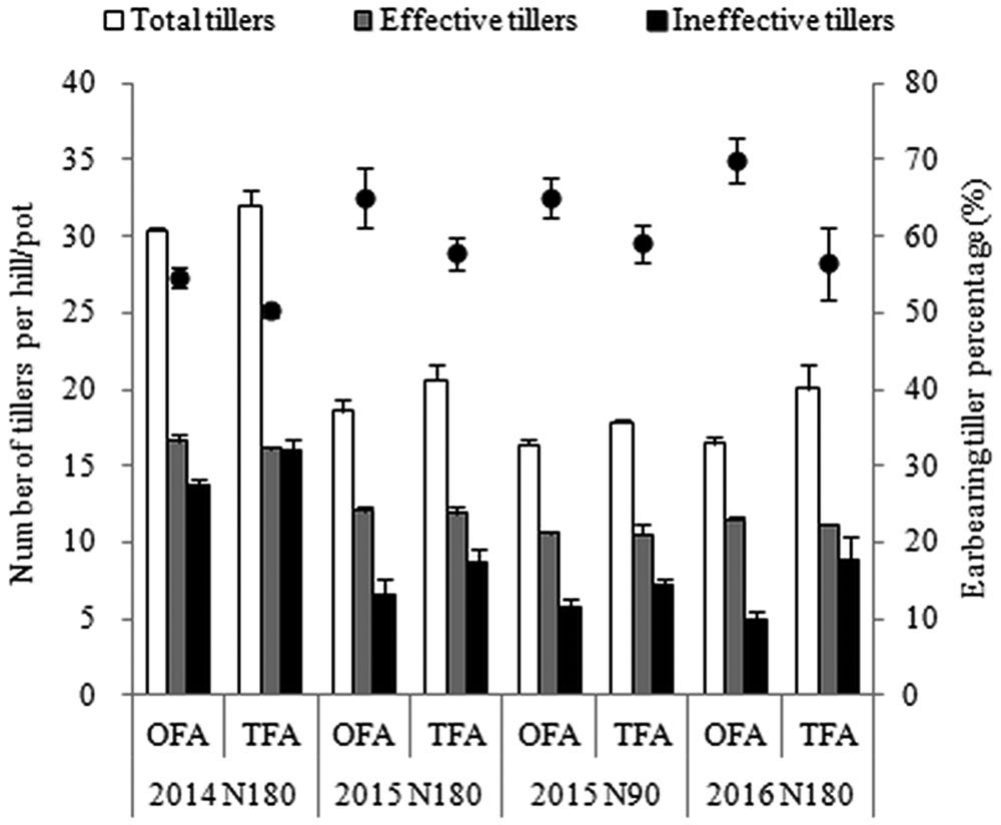
Effects of nitrogen applications on the number of tillers. N90: total nitrogen application rate of 90 kg ha^−1^; N180: total N application rate of 180 kg ha^−1^.

The number and the size of panicles are closely related to the growth of tillers, although moderate tillering contributes greatly to rice yields, excessive tillering leads to high tiller abortion, poor grain setting, and small panicle size, and further reduces the grain yield^17^. Results of three years’ experiments showed that OFA can reduce the emergence of ineffective tillers and increase the ear-bearing tiller percentage (Fig. 3). Compared with TFA, the number of ineffective tillers of OFA decreased by 13.7%, 23.2%, and 44.2% in the year of 2014, 2015, and 2016 respectively; and the ear-bearing tiller percentage under OFA condition increased by 8.6%, 11.4%, and 24% respectively. As a result, the number of effective tillers of OFA was a little more than that of TFA, and with less ineffective tillers, they could get more nutrients to grow stronger.

**Figure 4:**
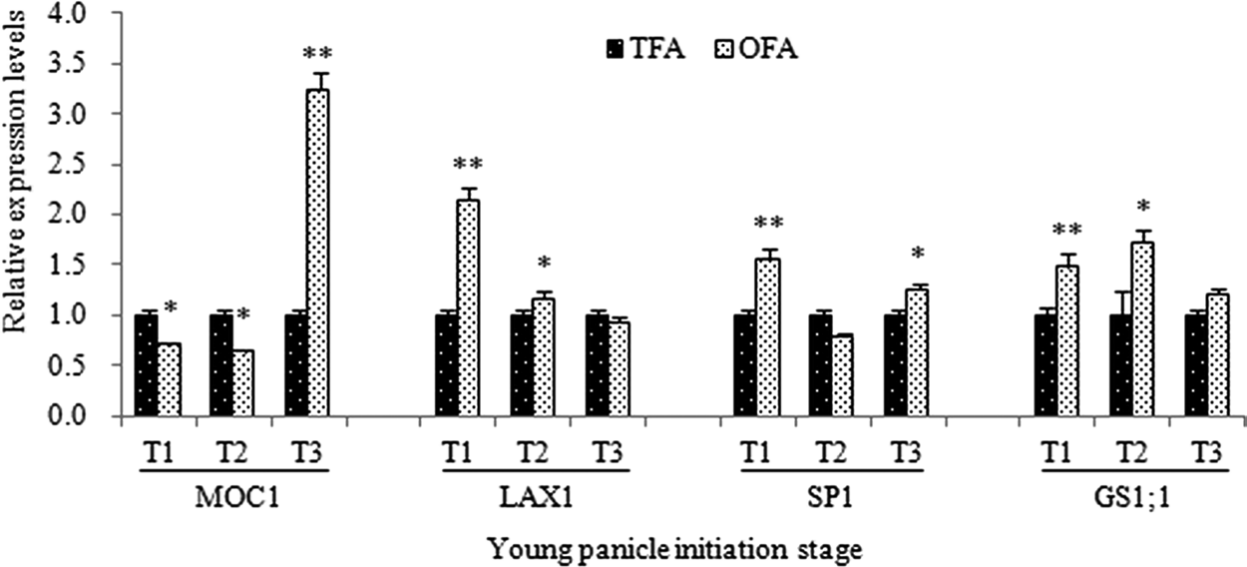
Relative expression levels of related genes. T1: differentiation stage of the first bract primordium; T2: differentiation stage of the primary branch primordium; T3: differentiation stage of the secondary branch and spikelet primordium; *MOC1*: *MONOCULM1*, *LAX1*:*LAX PANICLE1*; *SP1*: *Short Panicle1*; *GS1;1*: *Glutamine Synthetase1;1*; OFA: optimized nitrogen fertilizer application; TFA: traditional nitrogen fertilizer application; * and ** indicate significant difference of the two nitrogen treatments at 0.05 and 0.01 probability levels, respectively.

The expression levels of related genes were also examined. We explored the expression levels of *LAX PANICLE1* (*LAX1*) and *MONOCULM1* (*MOC1*) in three panicle differentiation stages (Fig. 4). These genes control the branches outgrowth^18,19^. The results showed that the expression level of *MOC1* in plants after OFA compared to that in plants after TFA was lower at the first bract primordium differentiation stage (T1) and the primary branch primordium differentiation stage (T2), but significantly higher (reaching as high as 3.3) at the secondary branch and spikelet primordium differentiation stage (T3). The expression of *LAX1* at T1 and T2 was higher in plants after OFA than in plants after TFA. Except for the number of secondary branches, the number of spikelets and panicle length in OFA treatment were increased significantly. We also determined the expression of *Short Panicle1* (*SP1*) and *Glutamine Synthetase1;1* (*GS1;1*), which are related to panicle size and spikelet number, respectively^18,20^. The results showed that the expression levels of *SP1* and *GS1;1* at T1, T2, and T3 in OFA treatment were higher than those in TFA treatment (expect for the expression of *SP1* in T2); the differences between the two treatments were significant or highly significant (expect for the expression of *GS1;1* in T3).

### Improvement of the photosynthetic area and net photosynthetic rate

**Figure 5:**
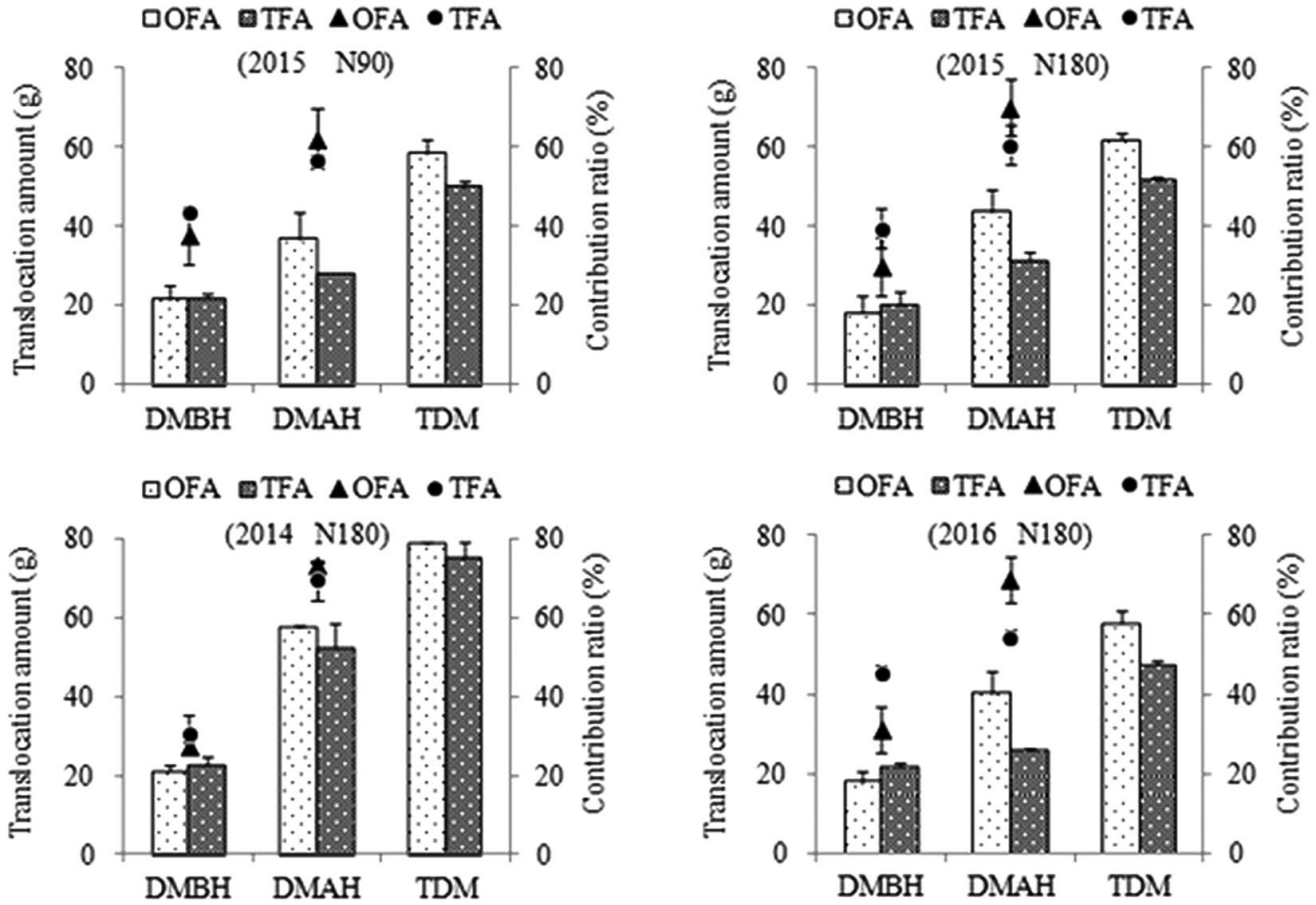
Effects of nitrogen applications on dry matter transportation. N90: total nitrogen application rate of 90 kg ha^−1^; N180: total N application rate of 180 kg ha^−1^; DMBH: dry matter production before heading; DMAH: dry matter production after heading; TDM: total dry matter accumulated; OFA: optimized nitrogen fertilizer application; TFA: traditional nitrogen fertilizer application.

The contribution ratio of photosynthates after heading in OFA and TFA treatments was 37.20% (24.61–46.01%) and 20.54% (8.81–38.81%) higher, respectively, than that of dry-matter production before heading (Fig. 5). These results indicated that dry-matter production after heading contributed more to grain yield of the *indica* hybrid rice, especially in OFA treatments. The leaf area, net photosynthetic rate, and foliar function period, especially for the upper three leaves, determined the capacity for dry-matter production after heading.

**Figure 6:**
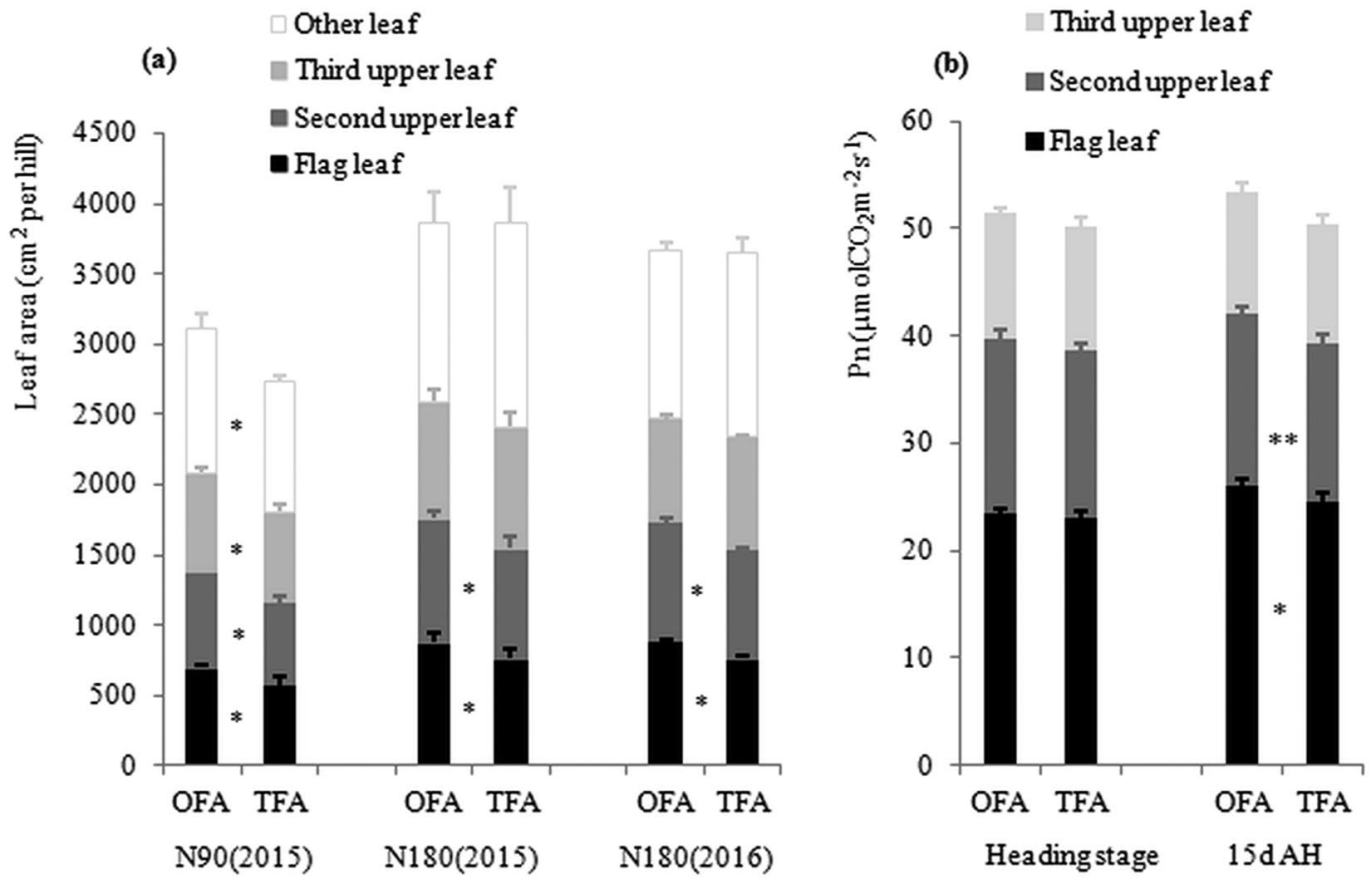
Changes in photosynthetic area and net photosynthetic rate (Pn) in response to different nitrogen applications. (**a**) leaf area; (**b**) net photosynthetic rate. OFA: optimized nitrogen fertilizer application; TFA: traditional nitrogen fertilizer application; N90: total nitrogen application rate of 90 kg ha^−1^; N180: total N application rate of 180 kg ha^−1^; AH: after heading stage; Pn: net photosynthetic rate; * and ** indicate significant difference of the two nitrogen treatments at 0.05 and 0.01 probability levels, respectively.

Consistent with the total effective leaf area, the high-effective leaf area, flag leaf area, and the second upper leaf area were larger in OFA than in TFA treatments; the flag leaf and second upper leaf area after OFA were significantly higher, by 18.43%, 11.89%, and 15.96%, 9.27% in 2015 and 2016, respectively, compared to those after TFA (Fig. 6a). A similar trend was found for the net photosynthetic rate of the upper three leaves in the heading stage and 15 days after heading—the net photosynthetic rate of the flag leaf was significantly higher than that of the second and third upper leaf, and the net photosynthetic rate after OFA was higher than that after TFA (Fig. 6b). These results indicated that the flag leaf was the main source of dry-matter production after heading, and the apparent increase in flag leaf area and the improved net photosynthetic rate denoted greater matter production ability under the OFA conditions.

### Extending the length of leaf function

The length of foliar function is influenced by many factors. Zhou *et al*.^21^ demonstrated that transferring too much of dry matter or too fast into panicle gives rise to premature leaf senescence, which adversely affects grain yield. Our previous study showed that postponing nitrogen application could increase chlorophyll content in rice leaves and delay leaf senescence^22^. The present study showed that the translocation amount and contribution ratio of dry-matter production before heading in OFA treatments decreased (Fig. 5), indicating that more matter was retained in the source, especially in green leaves, since the total amount of dry matter accumulated before heading was not different between the two nitrogen treatments.

**Figure 7:**
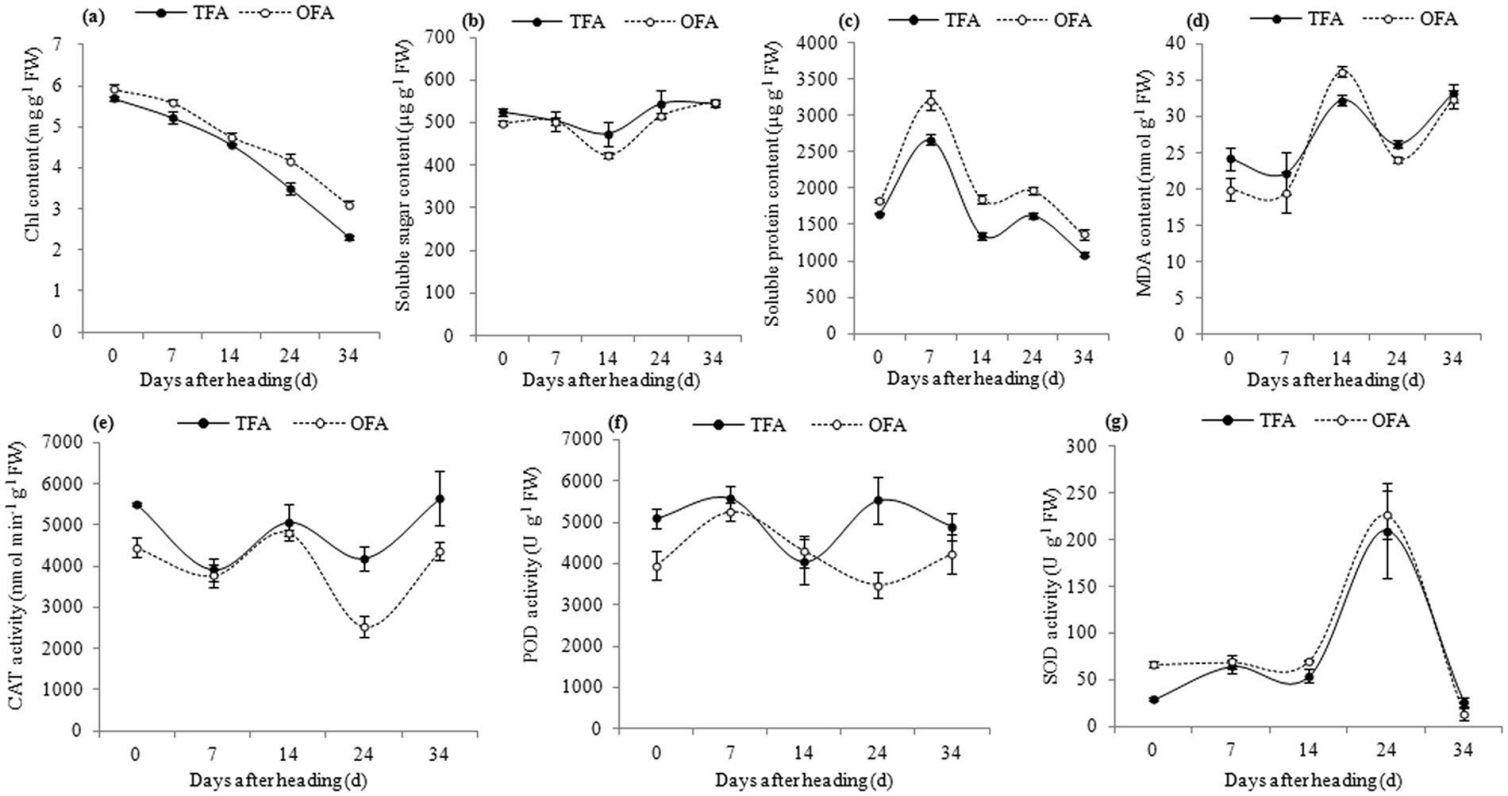
Physiological response to different nitrogen applications. (**a**) chlorophyll content; (**b**) soluble sugar content; (**c**) soluble protein content; (**d**) MDA content; (**e**) CAT activity; (**f**) POD activity; (**g**) SOD activity. Chl: chlorophyll; MDA: malondialdehyde; CAT: catalase; POD: peroxidase; SOD: superoxide dismutase. FW: fresh weight; OFA: optimized nitrogen fertilizer application; TFA: traditional nitrogen fertilizer application.

During leaf senescence, photosynthetic rates decline and leaf metabolism changes, affecting the content of chlorophylls, proteins, and sugars, and the antioxidases activities. The chlorophyll content gradually declined from the heading to maturing stage, although it remained higher in plants treated with OFA than in plants treated with TFA (Fig. 7a). The soluble sugar content decreased, reaching the lowest levels at 14 days after heading, and then increased gradually; the soluble sugar content was lower in OFA than in TFA treatment, although the difference between the two treatments was not significant until 34 days after heading (Fig. 7b). The contents of soluble proteins increased at a faster rate from the heading stage to seven days after heading and declined afterwards; they remained higher in plants treated with OFA than in plants treated with TFA after heading (Fig. 7c). With the advancement of leaf senescence in rice, the content of malondialdehyde (MDA) rose gradually until 14 days after heading, and it was lower under the OFA than under the TFA conditions (Fig. 7d). The activity of antioxidases, including catalase (CAT), peroxidase (POD), and superoxide dismutase (SOD), was altered with the senescence process moving forward, while little difference was observed between heading stage and 34 days after heading (Fig. 7e,f and g). Taken together, the OFA treatment resulted in lower activity of CAT and POD and higher activity of SOD compared to those in TFA treatment. Therefore, the reduction in translocated amount of dry matter produced before heading increased the chlorophyll and soluble proteins content and decreased the MDA and soluble sugar content from the heading to mature stage and extended the foliar function period, which together with the improvement in leaf area and net photosynthetic rate of functional leaves (Fig. 5a,b) made the promotion in dry matter production capacity after heading under the OFA conditions.

## Discussions

Our previous study have shown that tillers of different grade and leaf position affect significantly the growth and productivity of rice^23^. The results presented herein showed that OFA treatment decreased the occurrence of ineffective tillering, improved the ear-bearing tiller percentage, and promoted the growth of superior tillers (Fig. 3), which provides a stronger material foundation for development of functional leaves and young panicle in the late development stage. The survey of the heading stage showed that the high-effective leaf area was increased significantly (Fig. 6a). The vascular bundle in neck-panicle internode is the only way for nutrient transport from vegetative organs to reproductive organ. Liu *et al*.^14^ showed that the number of spikelets per panicle is positively related to the neck internode diameter. In the present study, the diameter of the panicle-neck internode and the number of large and small vascular bundles in the panicle-neck internode were increased after OFA (Fig. 1d,e and f), which has the potential to promote matter transport from source to sink and help the development of spikelets.

**Figure 8:**
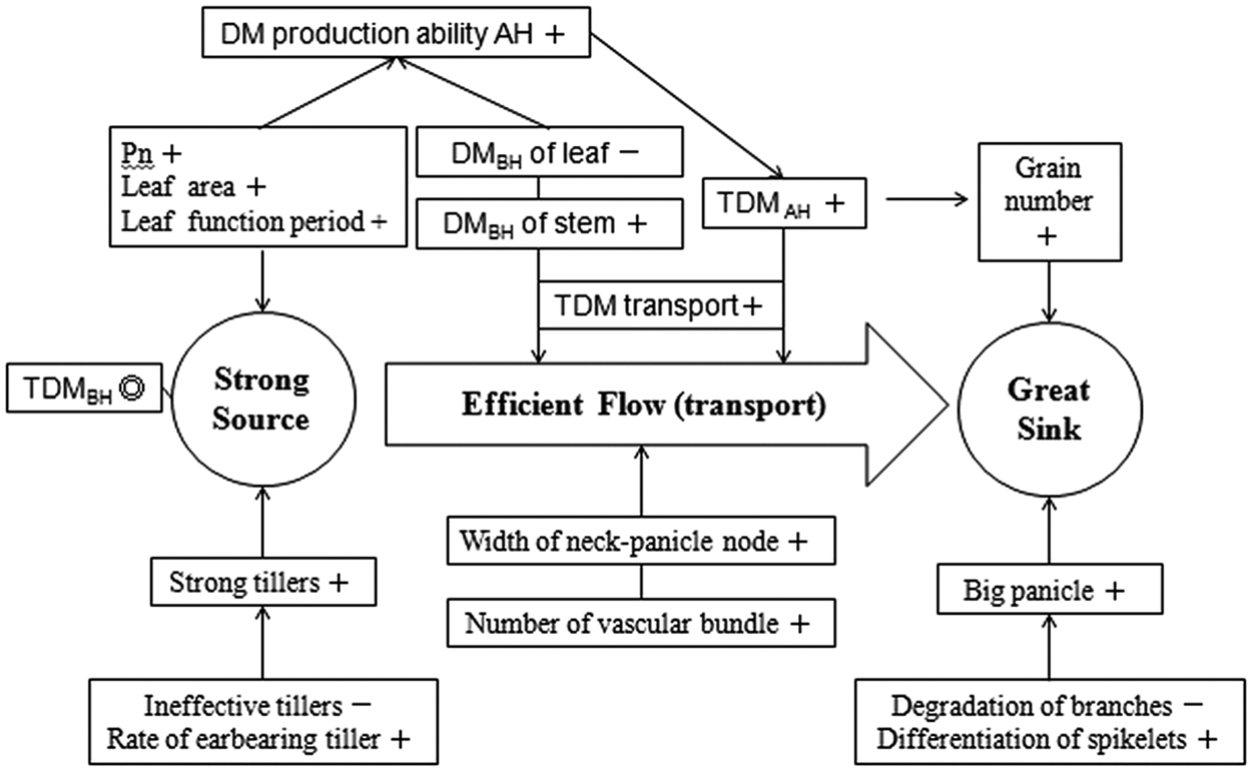
Yield-increasing mechanism as a consequence of postponed nitrogen application. AH: after heading; BH: before heading; Pn: net photosynthetic rate; DM: dry matter; TDM: total dry matter. The symbols “+”, “−”, “◎” represent increase, decrease, and little change, respectively.

The strength of the source, sink, and flow, and their harmonization determines the level of crop production. Rational cultivation management measures that will help to grow crops with strong source and sink and an efficient flow are an effective way to acquire high yield. The number of total spikelets and filled spikelets in plants after OFA was higher by 11.8% and 9.2%, respectively, than that in plants after TFA, resulting in bigger sink capacity. The increase in the number of vascular bundles and the diameter of the panicle-neck internode expanded the transport channel from source to sink and expedited the flux. The superior tiller growth with higher leaf area enhanced the source for grain filling. In conclusion, morphological structure and material basis changed under the condition of OFA rendered the source, sink, and flow stronger and more harmonized and consequently increased the rice yield (Fig. 8).

Photosynthesis provides the material basis for crop yield formation, and its production capacity is greatly influenced by leaf senescence. Leaf senescence is a complex developmental process that involves chloroplast breakdown accompanied by chlorophyll degradation and progressive loss of chloroplast, which causes leaf color changes from green to yellow^24^. During leaf senescence, a series of metabolic changes are inducted to allow recycling and remobilization of nutrients^25^, predominantly nitrogen. The present study showed that the decrease in chlorophyll content commenced at heading, while the content of soluble proteins decreased from seven days after the heading stage. Thus, the transfer of nitrogen lagged behind the chlorophyll degradation and the main stage for nitrogen transportation was focused mainly between seven and 14 days after heading. Chlorophyll degradation was slower under OFA conditions, and the content of chlorophyll remained at relatively high levels. Liang *et al*.^26^ reported that carbohydrate translocation from stems and sheaths to reproductive organs reached a maximum value at ten days after full heading. The content of soluble sugars in leaves reported herein fell to their lowest levels about two weeks after heading, and then gradually increased with leaf senescence, which indicated that carbohydrate translocation into stems and sheaths were more quickly than that in leaves, and carbohydrate translocation from vegetative organs to grains was mainly concentrated within two weeks after heading.

Takao *et al*.^27^ reported that accumulation of carbohydrates in leaves accelerated the senescence and the decline of the photosynthetic capacity, they also reported the negative relationship between carbohydrate content and photosynthetic rate observed in treatments with different nitrogen nutrition levels. So we can infer that the higher photosynthetic rate in OFA treatment in the later stage (14–34 days after heading) compared to that in TFA treatments was due to the content of chlorophyll and soluble sugars. Studies in barley (*Hordeum vulgare*) and *Arabidopsis thaliana* showed that nitrogen deprivation accelerates leaf senescence, but the supplementation with NO_3_^−^ at the start of senescence, halted or even reversed the senescence^28^. Rubisco content in rice remains high in plants given additional nitrogen fertilizer and indicates a close correlation between rubisco content and nitrogen levels in leaves^29^. Our earlier research has shown that the OFA management could significantly improve the nitrogen content in leaves from the heading to mature stage^11^, which may be the main reason for delayed leaf senescence and increased photosynthetic rate. Above all, to promote carbon transport and delay nitrogen transport from leaves to panicle after heading is likely to lead to a better photosynthetic productivity and yield, which may be the directions for future research.

The processes of plant aging are regulated by many factors, among which the injury caused by reactive oxygen species is the primary factor leading to leaf senescence^30,31^. Therefore, the reactive oxygen species scavenger system, especially the one that includes essential protective enzymes such as POD, SOD, and CAT, plays a pivotal role in hampering the production of intracellular reactive oxygen species^32,33^. MDA, a decomposition product of polyunsaturated fatty acids hydroperoxides and an important element of leaf senescence mechanism, may cause oxidative damage and therefore has been frequently used as a biomarker for lipid peroxidation^31,34^. During leaf senescence, MDA contents increase causing oxidative damage. In the present study, except at 14 days after heading, the MDA content in OFA treatments was lower than that in TFA treatments, indicating that the production of reactive oxygen species and the subsequent injury was lower under OFA conditions. Accordingly, the activity of reactive oxygen scavenging enzymes was lower compared to that in TFA treatment. These results showed that the protection mechanism under OFA conditions aimed to decrease the synthesis of reactive oxygen species and not to increase its scavenging once the damage has already been done. In conclusion, topdressing with nitrogen fertilizer at the jointing stage and 15–20 days after the jointing stage implemented in OFA management, increased the leaf chlorophyll content, delayed leaf senescence, improved the photosynthesis efficiency and thereby enhanced the matter production capacity, and ultimately increased the yield.

Our previous research also indicated that OFA treatment increases nitrogen uptake in panicle and improves nitrogen use efficiency^11^. These properties are dependent on the absorption, transportation, and remobilization of nitrogen. Other studies have demonstrated that *SP1* is related to the transport of nitrogen-containing substrates and determines rice panicle size^18,35,36^, whereas *GS1;1* plays a role in nitrogen remobilization during senescence and determination of grain number, grain size, and grain filling^20^. Hence, the upregulated *SP1* and *GS1;1* in plants after OFA not only improved the yield, but also affected the nitrogen utilization increment. Therefore, supplemented nitrogen determines the expression of *SP1* and *GS1;1*, which in turn control the yield and regulate nitrogen utilization. There is little difference in the number of differentiated secondary branches between OFA and TFA treatments; the OFA treatment decreases significantly the number of degenerated secondary branches thus increasing the number of survived secondary branches^12^. Therefore, the number of differentiated secondary branches is a consequence of the combination of downregulated *MOC1* and upregulated *LAX1* at T1 and T2, while the significant upregulation of *MOC1* at T3 may be the main reason for the decrease in degenerated secondary branches. The percentage of degenerated secondary branches was very high in *indica* hybrid rice cultivars, and a full understanding of the regulatory mechanisms of genes (i.e., *MOC1*) that could decrease the degradation of secondary branches will help to increase the number of spikelets and ultimately lead to higher yield.

## Materials and Methods

### Experimental design

The experiments were conducted in paddy fields at Wenjiang (30°43′ N and 103°52′ E), Sichuan, China, during the rice growing seasons in 2014–2016. The characteristics of the soil are described in Table 1. The cultivar Fyou498 (F32A × Shuhui498), a main popularized variety, was used as test plant. The experiment in 2015 was performed using a two-factor randomized block design with two nitrogen treatments and two nitrogen application rate. The two nitrogen treatments were: traditional nitrogen fertilizer application (TFA; base and tillering fertilizer in proportions 70% and 30%, respectively; accepted by Chinese farmers), and OFA (35% applied at base, 15% at early tillering stage, 25% at jointing stage, and 25% at 15–20 days after jointing stage). Nitrogen application rate were 180 and 90 kg ha^−1^. A single factor and two-level (TFA and OFA with the same rate of 180 kg ha^−1^) experiment with randomized block design were conducted in 2016. Other fertilizers, 90 kg P_2_O_5_ ha^−1^ and 180 kg K_2_O ha^−1^, were applied in all treatments. The transplanting density was 15 × 10^4^ hills ha^−1^. The 20-cm-high ridges were built between the plots and coated with film to prevent leaching of fertilizer and water into adjacent plots. The study conducted in 2014 was performed as a pot experiment, laid out in a single factor randomized block design, with the same nitrogen treatments (TFA and OFA). The two nitrogen applications had the same total nitrogen amount of 1.2 g pot^−1^ (equal to 180 kg ha^−1^ applied to the paddy with a density of 15 × 10^4^ hills ha^−1^). Other fertilizers were applied at a rate of 2.4 g P_2_O_5_ pot^−1^ and 1.2 g K_2_O pot^−1^ in all treatments. Each pot was filled with 12.0 kg paddy topsoil that was air dried and thoroughly mixed. Each treatment consisted of 22 pots, with three replicates. The pots were placed in a greenhouse to prevent washing out of applied fertilizers by rain. Seedlings were sowed on 18 April and transplanted on 28–31 May at a rate of two seedlings per hill or per pot at the same spacing. Other rice management practices were similar to those applied to the paddy field.

**Table 1:** Soil characteristics in the experiments.

Year	pH	Organic matter (g kg^−1^)	Total N (g kg^−1^)	Total P (g kg^−1^)	Total K (g kg^−1^)	Alkali-hydrolyzale N (mg kg^−1^)	Available P (mg kg^−1^)	Available K (mg kg^−1^)
2014	7.26	29.17	1.58	0.38	3.91	116.51	13.02	65.04
2015	5.80	29.58	1.28	0.50	7.75	97.64	20.27	56.52
2016	5.70	30.74	1.27	0.54	8.85	100.75	26.39	28.97

### Indexes and measurement methods

#### Tiller growth and decline

After rice transplanting in 2014–2016, the number of tillers were measured from 20 hills (all pots for pot experiment) every seven days until the full heading stage, before harvesting, the number of effective panicles per hill was determined from 60 hills (all pots for pot experiment) of each plot. Then the number of total tillers emerging, effective tillers and ineffective tillers were determined.

#### Dry matter determination

Six hills or pots with the average number of tillers (determined from sixty hills or pots, the same below) in each treatment were collected as samples at the heading and maturing stages in 2014–2016. The samples were divided into leaf, stem and sheath, and panicle, exposed to 105 °C for 60 min, and then dried at 75 °C to constant weight. The dry matter of each part was determined.

### Leaf area and net photosynthetic rate

Six hills with the average number of tillers in each treatment were collected as samples at the heading stage in 2015 and 2016. The length and maximum width of each green leaf were measured with a ruler. Leaf area was calculated by multiplying the coefficient (0.75) by the leaf length and maximum width. The net photosynthetic rates of the flag leaf, the second upper leaf, and the third upper leaf were determined using an LI-6400XT Portable Photosynthesis System (LI-COR, Inc., Lincoln, NE, USA) in 2016. The measuring conditions and methods are described in Wang *et al*.^37^.

### Panicle morphology and composition

Fifteen hills with the average number of effective panicles in each treatment were collected as samples at the mature stage. The number of secondary branches (in 2014), the number of filled and unfilled spikelets, the length of panicles(in 2014–2016), and the diameter of the neck-panicle node (in 2015 and 2016) were measured for each panicle. Fresh segments of primary stems eared consistently were cut about 1 cm below the panicle base node. After fixation and desilication, the stems were made into paraffin slice with safranine and fast green staining to observe the number of vascular bundles under a microscope and photographed (in 2015 and 2016).

### Gene expression in panicle and physiological indices in leaf

Fresh samples for gene expression were collected from 15 hills per plot at three panicle initiation stages (in 2016), the first bract primordium differentiation stage (T1), the primary branch primordium differentiation stage (T2), and the secondary branch and spikelet primordium differentiation stage (T3). Ten flag leaves from each plot were collected from heading to mature stage and used to determine physiological indices (in 2015), including the content of chlorophyll, malondialdehyde (MDA), soluble sugars, and proteins, and the activity of catalase (CAT), peroxidase (POD), and superoxide dismutase (SOD). All samples were treated with liquid nitrogen immediately after collection and then stored at −80 °C until use. RNA extraction and quantitative RT-PCR were conducted as described previously^38^. The primers for quantitative RT-PCR analysis of *LAX PANICLE1* (*LAX1*), *MONOCULM1* (*MOC1*), *Short Panicle1* (*SP1*), and *Glutamine Synthetase1;1* (*GS1;1*) expression were presented in the Table 2. The physiological indices were measured by microdetermination using the kits (Suzhou Comin Biotechnology Co., Ltd., Shuzhou, Jiangshu, China) for each index following the manufacturer’s protocol.

**Table 2:** The primers for quantitative RT-PCR analysis of genes.

Genes	Forward primer	Reverse primer
*MOC1*	TCTCGCCTTCCTGTCCATTG	CCACCTGCACCCCATCATTA
*LAX1*	TTGCCTTGCTGTGTCTTCGT	GACTCCTCACCGTGGACATT
*SP1*	TCCAGGCGTTCGAGATGATG	TTTGACAGCGGGTAGTGCAT
*GS1;1*	CACCAACAAGAGGCACAATG	ACTCCCACTGTCCTGGCAT

*MOC1: MONOCULM1, LAX1: LAX PANICLE1; SP1: Short Panicle1; GS1;1: Glutamine* Synthetase1;1.

### Ethical approval and data availability statement

These field studies did not involve any endangered or protected species; no specific permits were required for the described field studies, and the landowner (Rice Super-high-yield Production Model Base) permitted these field studies to be carried out. The methods were carried out in accordance with the relevant guidelines and regulations, and all data in this paper would be available after publication.
